# Modelling trade war between two countries under the international division of Labour

**DOI:** 10.1016/j.heliyon.2024.e24633

**Published:** 2024-01-24

**Authors:** Chun-Hung Chen

**Affiliations:** Department of Accounting, Chaoyang University of Technology, 168, Jifeng E. Rd., Wufeng District, Taichung, 413310 Taiwan

**Keywords:** Trade war, International division of labor, Tariff, Import trade, Export trade

## Abstract

This study aims to determine the trigger conditions behind trade wars and explore the prerequisites required to institute trade subsidies. Additionally, it investigates the potential effectiveness of both a trade war and a trade subsidy within an economic framework shaped by the international division of labor, particularly focusing on intermediate goods production. The study formulates a theoretical model for trade wars and derives three crucial findings. Firstly, it establishes that a critical condition for triggering a trade war hinges on maintaining a balanced self-price coefficient of demand, neither excessively large nor small relative to the cross-price coefficient of demand. Secondly, failure to meet this condition would preclude the occurrence of a trade war; instead, it would prompt the emergence of trade subsidies and reciprocal actions between the two countries involved. Lastly, the scale of the trade war or a trade subsidy in this study is not influential in the countries of trade war. Therefore, this study recommends that changing the current market structure of the international division of labor would be necessary to make the trade war or trade subsidies effective.

## 1 Introduction

Existing studies extensively analyze competitiveness of international goods; however, there remains a notable dearth of discussions regarding complementarity within the contemporary international division of labor. Therefore, the theoretical investigation aiming to complement the complete complementarity within the international division of labor serves as the primary research motivation for this study.

Therefore, this study focuses on the upstream industries of three countries where trade demonstrates complementarity. The intermediate goods industries in these three countries exhibit mutual complementarity, while the downstream industry products are exported to a competitive market in a fourth country.

The central question of this research revolves around the following question. Given the prevailing phenomenon of the international division of labor on intermediate goods worldwide, does the efficacy of a trade war persist? Within this framework of international division of labor, this study intends to assess the effectiveness of a trade war if it proves effective under such circumstances. Conversely, if the trade war appears ineffective, the study posits discarding the premise of the international division of labor to render the trade war effective. This study aims to analyze trade policies through three primary lenses: the international division of labor, the interdependence of intermediate goods produced by firms in three countries, and the impacts of tariff-based trade wars. Employing a theoretical model, this research seeks to delineate the strategic direction of trade, confirming the requisite conditions for triggering a trade war. More specifically, by analyzing and comparing company competitiveness and societal welfare of nations, this study aims to formulate effective international trade policies that benefit all countries involved in potential trade wars. The underlying methodology of this study adopts an analytical research approach, using theoretical models to simplify the complexities of real-world phenomena and elucidate the international trade policies pertinent to the international division of labor.

The onset of trade wars was the result of geopolitical competition and the surge of economic nationalism that began in 2014. This period witnessed trade wars between various nations, including the United States and China, Japan and South Korea, the United States and Russia, among others.

On March 22, 2018, President Donald Trump signed a memorandum imposing tariffs on commodities imported from China, sparking a US-China trade war. The trade volume between the United States and China amounts to more than $550 billion. Both nations continue to impose significantly high tariffs on imports as a response to perceived unfair trade practices and violations of intellectual property rights. However, the existing literature on theoretical models regarding the US-China trade war remains deficient. To bridge this gap, this study introduces a model aimed at augmenting the theoretical underpinnings of the trade war. The ensuing analysis yields crucial policy implications and proposes international trade remedies applicable to all nations.

To initiate the identification of gaps in previous studies, our primary focus will be on the context of trade wars. Specifically, our objective is to examine the use of the international division of labor in the literature encompassing the import and export of intermediate and final goods among nations.

Mihaylov and Sitek (2021) [35] discovered that the present interaction between certain nation-states has progressively transitioned into trade wars, forming a hybrid convergence of geopolitics and geoeconomics.

Fajgelbaum and Khandelwal (2022) [19] employed a theoretical model to analyze and discovered that starting in 2018, the United States began a trade war with China. At the end of 2019, the United States imposed tariffs of approximately $350 billion on Chinese imports, while China retaliated against US exports, levying tariffs of approximately $100 billion. Their model diverges from ours, as their study does not delve into the industrial structure of the international division of intermediary goods.

Li, He, and Lin (2018) [32] used an abstract theoretical framework to analyze the US-China trade war. Their study revealed that, when comparing the impact of the trade war between the two nations, the United States incurred fewer losses than China. Additionally, the US-China trade war is expected to significantly harm numerous countries and the global economy, notably affecting GDP and manufacturing employment. Their model diverges from ours, as their study does not dive into the industrial structure of the international division of labor among intermediary goods.

Jung (2021) [28] discovered that as the intensity and duration of the US-China trade war escalate, it will significantly affect the global economy. In particular, for nations highly dependent on the economies of the United States and China, the reduction in the number of firms exporting from these two countries to third-party nations has been most pronounced within the transportation industry.

Zeng, Wells, Wilkins, and Gu (2022) [46] analyzed the impact of bilateral tensions on US imports from China between 2002 and 2019. Their findings suggest that potential “sunk costs” might not be adequate to mitigate the US-China trade war.

Dhar, Le, Coffelt, and Shaturaev (2023) [16] observed that factors such as the US-China trade war and globalization contributed to the ongoing decline in China's international competitiveness within labor-intensive industries. China's competitiveness saw a sustained downward trend through the latter part of 2020, while Vietnam's competitiveness exhibited a continuous rise.

Benguria, Choi, Swenson, and Xu (2022) [5] observed that the US-China trade war resulted in a rapid rise in import and export tariffs encountered by Chinese companies, ushered in an era characterized by trade policy uncertainty (TPU).

Shin and Balistreri (2022) [39] used a theoretical model to analyze the trade war. They examined the onset of a new trade war between South Korea and Japan in mid-2019. Their analysis revealed Japan's implementation of export controls, countered by South Korea's adoption of a boycott against Japanese goods. The study revealed losses of $1 billion for South Korea and $346 million for Japan. Their model diverges from ours as their study encompasses nontariff trade disputes and does not delve into the industrial structure of the international division of intermediary goods.

Toraubally (2022) [43] employed a theoretical model in their analysis, focusing on the relationship between the traditional Ricardo-Haberlerian (1817; 1936) [[22], [36]] theory of comparative advantage (RTCA) in trade wars. Their thesis echoes similarities with our findings, indicating the incompatibility between comparative advantage and trade wars. However, their model diverges from ours, as their study does not delve into the industrial structure of the international division of labor among intermediary goods.

Zheng, Zhou, Li, Padula, and Martin (2022) [47] investigated the economic repercussions of the trade war during the Trump administration, specifically focusing on the increased tariffs imposed by the United States and China. The United States increased tariffs on China's industrial goods by a factor of six, with substantial increases observed in tariffs on intermediate and capital goods. On the contrary, China augmented tariffs on American agricultural products by more than fivefold. The US-China trade war has resulted in a decrease in import volumes, with a reduction of 4.9 % in China and 4.5 % in the USA.

Sun, Luo, and Zhou (2022) [42] used theoretical models for analysis and revealed that amidst the looming threat of trade wars and antilocalization trends, countries have entered sequentially into bilateral and multilateral trade agreements. Their study also indicated that China's participation in these trade pacts would promote a more balanced development between the regions of China. However, their model diverges from ours, as their study does not delve into the industrial structure of the international division of labor concerning intermediary goods.

Chen (2023) [14] investigated strategies to improve the profitability of domestic upstream companies within the international input market amidst a trade war, specifically exploring the impact of imposing tariffs on imported final goods. Upon implementing input export restrictions (IER), the study revealed a positive correlation between the tariff rate and the input prices. This correlation facilitated an increase in profits for both domestic upstream and downstream companies.

Ahn, Greaney, and Kiyota (2022) [2] examined the trade war between South Korea and Japan, specifically delving into the boycott of Japanese travel by Korean consumers in 2019 to assess the repercussions of the trade war. Their findings indicated that the export losses incurred by Japanese regions highly dependent on Korean tourists before the boycott surpassed those experienced by regions lowly dependent on Korean tourists before the boycott.

Latipov, Lau, Mahlstein, and Schropp (2023) [31] examined the trade war between the United States and Russia. Alongside the current import restrictions on energy commodities such as oil, natural gas, and coal, the United States recently introduced a revised tariff framework, imposing increased import duties on 570 Russian goods. The economic repercussions of the trade war between these nations vary across the sectors targeted by the United States.

Ye, Shen, Golson, Lee and Li (2022) [45] determined the temporal duration of each significant event by monitoring the keyword “trade war” and concluded that the influence of the trade war gradually diminishes. Furthermore, downstream and midstream companies experienced notably greater impacts compared to their upstream counterparts.

Chen and Kuang (2023) [15] used a theoretical model to scrutinize trade wars, establishing a trading game within an endogenous time series framework to explore potential trade wars in a vertical bilateral trade model. Within a fixed-time game scenario, their analysis revealed that the pricing disparity in intermediate goods stems from variations in the production costs of final goods. When the cost differential reaches a significant threshold, both nations are incentivized to initiate a trade war, resulting in a game of multiple equilibrium. Although their model shares similarities with ours, their study diverges by not discussing the industrial structure of the international division of intermediate goods and they presume homogeneity for both intermediate and final goods produced across different countries.

Given the escalating landscape of protectionism, the imminent severity of international trade wars requires an examination of the existing industrial model within the international division of labor. This analysis aims to elucidate the potential repercussions of the growing wave of trade conflicts around the world. Moreover, it aims to deliberate on strategies aimed at mitigating the plausible aftermaths of trade wars by orchestrating alterations within the industrial framework.

The article conducts a comparative analysis with the existing literature. Over the past more than three decades, the evolution of the new trade theory, often referred to as strategic trade theory, has been notable. Scholarly discourse has progressively broadened, transitioning from trade involving only final goods, as evidenced by seminal works like Brander and Spencer (1985) [11] and the classic study by Eaton and Grossman (1986) [18], to encompass trade involving intermediate goods. Notable studies involving trade exclusively in final goods include Brander (1981) [8], Brander and Krugman (1983) [9], Dixit (1984) [17], Brander and Spencer (1984) [10], Hwang (1984) [24], Harris (1985) [23], Venables (1985) [44], Mai and Hwang (1988) [33], and Chen (2016) [13]. Those exploring intermediate goods trade encompass Salop and Scheffman (1983, 1987) [37,38], Jones and Spencer (1989) [27], Spencer and Jones (1991, 1992) [40,41], Chang and Chen (1994) [12], Bernhofen (1996, 1997) [6,7], Ishikawa and Lee (1997) [25], Ishikawa and Spencer (1999) [26], Lee and Wong (2005) [30], and Chen (2016) [13]. Strategic trade theory assigns dual roles to intermediate goods trade. First, it involves optimizing the trade policy for intermediate goods. Second, it impacts the final goods production methodology, influencing therefore the optimal trade policy for these final goods. This study deviates from the aforementioned literature by considering the interrelationship between final goods and intermediate goods in international trade, with a specific focus on trade wars.

The work of Golovko and Sahin (2021) [20] presented an intriguing perspective on international trade theory. They employed the theoretical model and extended it into empirical studies of international trade. A commonality between their paper and this study lies in their foundations in theoretical models. Their work used the theoretical gravity model to elucidate international trade dynamics, whereas this study operates within the realm of the theoretical model of international division of labor to explain international trade. The divergence between their paper and this research becomes apparent as the former extended the established gravitational theoretical model, adapting it into an empirical framework to delve into international trade discussions. On the contrary, the latter remains a purely theoretical model focusing on the study of international trade wars.

Cournot's (1838) [4] oligopolistic model stands as a seminal theoretical framework to understand complementarity. Cournot specifically introduces the copper-zinc duopoly, a distinctive model in which one firm exclusively produces zinc while the other firm exclusively produces copper. In particular, in this model, zinc and copper are not considered final goods, but are indispensable elements that form brass, which is the final goods or ‘system goods’. On the other hand, Matutes and Regibeau (1996) [34] and Katz and Shapiro (1994) [29] presented comprehensive theoretical models centered on complete complementarity. Their analyses delved into the selection of the compatibility, standardization, and network externalities of a firm. While their contributions are significant within the literature, their focus does not encompass the international division of labor. Consequently, our study regards the international division of labor among three countries as a horizontal division and introduces a model analysis rooted in the tariff war arising between two specific countries.

The advantages of this study in theory, research methods, and argument are described below. First, the theoretical advantage of the study lies in its adoption of the concept that intermediary goods constitute the international division of labor, which forms the basis for the analysis of the trade war of final goods. This approach allows the theory to align with the current situation of contemporary international trade. Second, the methodological advantage of the study lies in its dependence on theoretical model analysis rather than empirical research, which ensures that conclusions remain independent of data fluctuations. Third, the argumentative advantage of the study lies in the potential of the conclusions derived to serve as guiding references for countries in shaping their international trade policies.

The research contribution of this study has forged a theoretical framework for understanding international trade wars, serving as a foundation for subsequent research and further empirical analysis. The primary research contributions and findings of this study can be summarized as follows.

The results of this study indicate that within an international division of labor on the intermediate goods market, the demand function for final goods plays a pivotal role in determining the occurrence of a trade war. Specifically, the presence of a trade war hinges on the self-price coefficient of demand and the cross-price coefficient of demand. Failure to meet these conditions will likely incite a trade war, while compliance may result in trade subsidies. Furthermore, regardless of whether a trade war ensues, variables such as social welfare, taxation, profits from upstream and downstream firms, prices of intermediate and final goods, and the quantity of exports remain unaffected by the scale of the trade war or subsidies. Therefore, modifying the existing market structure within the international division of labor has the potential to trigger effectiveness in trade wars or subsidies.

The structure of this study is described below. This section serves as the introduction. Section 2 establishes a theoretical framework and analysis that cover six companies in three countries. It delves into the equilibrium when governments adopt trade war policies in a scenario where intermediate goods demonstrate complete international complementarity and where each company's intermediate goods manufacturers have a monopoly. Section 3 summarizes the conclusions drawn from this study.

## 2 Theoretical framework and analysis

The theoretical model used in this study is founded on the perspective of Professor M. Friedman from the University of Chicago, who emphasized that the quality of an economic model is not determined by the realism of its assumptions, but by its capacity to elucidate phenomena and predict future outcomes. Therefore, despite being perceived as subjectively orientated, this model still holds substantial practical reference. Essentially, this research constructs the model based on certain hypothetical variables and functions, yet rigorously demonstrates its derivation through mathematical software verification, omitting unnecessary computational steps from the text.

The existing literature utilising theoretical models to analyze trade wars includes several notable contributions. Li, He and Lin (2018) [32] used a multicounty global general equilibrium (GE) model to numerically simulate the potential economic impact of a US-China trade war. Audzei and Bruha (2022) [3] constructed a dynamic stochastic general equilibrium model that includes the euro area, the United States, and China to examine the US-China trade war. Shin and Balistreri (2022) [39] used a multiregional general equilibrium model to analyze the impact of the trade war between Japan and South Korea. Toraubally (2022) [43] used optimization problems and logic to illustrate that trade is influenced by strategic considerations. Sun, Luo, and Zhou (2022) [42] developed theoretical and empirical models to examine whether regional trade agreements could improve the quality of exported products amid uncertainties from the trade war. Chen and Kuang (2023) [15] formulated a theoretical game within an endogenous time series, investigating potential trade wars in a vertical bilateral trade model. Although our theoretical model closely aligns with theirs, crucial disparities define our research from that of Chen and Kuang (2023) [15]. The primary distinctions encompass: (1) Our study delves into the industrial structure of the international division of intermediate goods, whereas the latter does not. (2) We assume complete complementarity among intermediate goods produced by different countries, whereas the latter assumes homogeneity among these goods. (3) Our study assumes partial heterogeneity among final goods produced in different countries, while the latter assumes homogeneity. (4) We incorporate three countries to clarify the impact of the trade war between two of them, whereas the latter assumes only two countries.

The objective basis supporting the construction of the series of mathematical models is similar to that of Chen and Kuang (2023) [15]. The subsequent mathematical model will be lucidly articulated, precisely defined, and rigorously expounded.

**Fig. 1:**
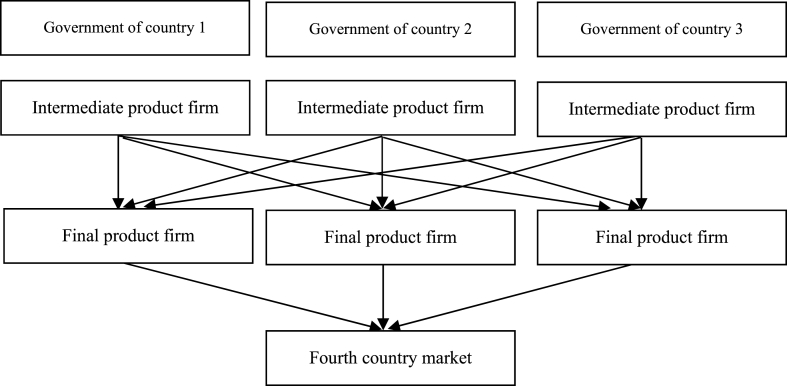
Theoretical model framework for tariff trade war.

Fig. 1 presents the theoretical model. First, it establishes a model featuring heterogeneous goods in four countries and six firms. The model encompasses 10 participants: the governments of Country 1, Country 2, and Country 3; upstream industries (intermediate goods firms) in Countries 1–3; downstream industries (final goods firms) in Countries 1–3; and a consumer (Country 4). The governments of the three producing countries independently determine an optimal trade policy for exports. In each country, there exists an intermediate goods company and a final goods company. Intermediate goods firms have a monopoly on the production of key intermediate goods in their respective countries. Additionally, the three final goods firms each acquire one unit of intermediate goods from each of the three intermediate goods firms. This single unit of intermediate goods is crucial in producing a unit of final goods. A unit of intermediate goods is used to produce a unit of final goods. In other words, each final goods firm purchases one unit of intermediate goods from each of the three intermediate goods firms to manufacture one unit of the final goods. The final goods produced by the three final goods firms are sold in country 4. The final goods produced by these firms are then sold within Country 4. These three final goods are heterogeneous, and consumers within the producing countries do not assume consumption of these final goods. To accentuate the impact of the trade war, this study assumes that Country 3 practices free trade, that is, no trade tariffs or subsidies are imposed on Countries 1 and 2. On the contrary, Countries 1 and 2 impose trade tariffs on each other. The rationale for the inclusion of three countries in this model is rooted in the contemporary international division of labor, which involves more than just two countries. For example, Jung (2021) [28] mentioned that South Korea is highly dependent on the United States and China in terms of economy and has the phenomenon of international division of labor. Zeng, Wells, Wilkins, and Gu (2022) [46] also mentioned that the US-China trade war affects industries in other countries that have high global value chain connections with China.

The model is structured in three stages. In the first stage, the governments of the three producing countries intervene in the export of final goods and may opt for export taxation, free trade, or export subsidies. However, the WTO prohibits export subsidies due to their perceived hindrance to international competition. Therefore, the model reasonably assumes the absence of export subsidies for final goods. The governments of countries 1 and 2 intervene in the import of intermediate goods through tariffs or subsidies. Simply put, all three governments adopt trade policies for final good exports by domestic firms, and countries 1 and 2 do so for foreign import firms. This means that governments impose a quantity tax or a quantity subsidy on imports. The government of country 1 imposes tariff $t_{12}$ on the import of intermediate goods from country 2, and the government of country 2 imposes a tariff of $t_{21}$ on the import of intermediate goods from country 1. This model assumes that country 3 follows a free-trade policy, that is, no import tariff is imposed on intermediate goods from countries 1 and 2. Furthermore, it assumes that the import tariffs imposed on the intermediate goods firms in countries 1 and 2 can be passed on to the final goods firm. Therefore, the cost of final goods is the sum of the original prices for intermediate goods in country i (i=1,2,3), $a_{i}$, and the tariff, $t_{ji}$ (i≠j), and the net income per unit of the intermediate goods firm ($a_{i}+t_{ji})-t_{ji}$ is estimated at original price $a_{i}$. The export tax levied by the governments of countries 1, 2, and 3's governments on final goods are $s_{1}$, $s_{2}$, and $s_{3}$, respectively. If tariff rates $t_{12}$ and $t_{21}$ are a positive number, it is considered an import tariff. But, if they are negative, it is an import subsidy. Similarly, it is an export tariff if tax rates $s_{1}$, $s_{2}$, and $s_{3}$ are positive and free trade of export if they are zero. This is because export subsidies are prohibited by the WTO as per the Agreement on Subsidies and Countervailing Measures (SCM), it is only possible to impose export tariffs or to have free trade. Thus, this study assumes $s_{1}$, $s_{2}$, $s_{3}$ ≥0. Since the social welfare of the producing country is the sum of the profits of upstream and downstream firms and the tax revenue of governments. The objective function of the governments of the three countries can be written without loss of generality. The objective function of the government of the i-th country (i=1,2,3) is equation (1).

(1) $$W_{i}=T_{i}+\Omega_{i}+\Pi_{i}$$

where $T_{i}$ is the tax levied by the government of country i, $\Omega_{i}$ is the profit earned by intermediate goods firm in country i, and $\Pi_{i}$ is the profit of the final goods firm in country i. $T_{i}$ is estimated as follows (i.e., equations (2), (3), (4)):

(2) $$T_{1}=s_{1}q_{1}+t_{12}q_{1}$$

(3) $$T_{2}=s_{2}q_{2}+t_{21}q_{2}$$

(4) $$T_{3}=s_{3}q_{3}$$

where $q_{i}$ denotes consumer demand in country 4 for the final goods of country i (i=1,2,3).

In the second stage, intermediate goods firms in the three countries monopolize the production of intermediate goods and set prices $a_{i}$ to maximize their own profits. For simplicity, the production costs of intermediate goods firms are assumed to be zero except for the purchase of intermediate goods. Since there is an indirect demand for intermediate goods by downstream industries, the profit function of intermediate goods firms in country i is equation (5).

(5) $$\Omega_{i}=a_{i}\left(q_{1}+q_{2}+q_{3}\right)$$

Known from the above, the first stage sets the tone for the tariff trade war. Country 1 imposes a high tariff of $t_{12}$ on imports from country 2 of intermediate goods, and country 2 imposes a high tariff of $t_{21}$ on the imports of intermediate goods from country 1.

In the third stage, the final goods firms in the three countries combine the three intermediate goods in equal proportion to form the final goods. Each unit of intermediate goods can be combined into one unit of final goods. The final goods firms engage in price competition in country 4 and set price $p_{i}$ to maximize their own profits. For the sake of simplicity, the combined cost of the final goods firm is set to zero, so it only incurs the cost of purchasing intermediate goods. Consequently, the profit functions of the final goods firms in the three countries are as follows (i.e., equations (6), (7), (8)):

(6) $$\Pi_{1}=\left(p_{1}-s_{1}\right)q_{1}-{\left[a_{1}+\left(a_{2}+t_{12}\right)+a_{3}\right]q}_{1}$$

(7) $$\Pi_{2}=\left(p_{2}-s_{2}\right)q_{2}-{\left[\left(a_{1}+t_{21}\right){+a}_{2}+a_{3}\right]q}_{2}$$

(8) $$\Pi_{3}=\left(p_{3}-s_{3}\right)q_{3}-{\left[a_{1}{+a}_{2}+a_{3}\right]q}_{3}$$

The demand function for the final goods in country 4 is derived from optimized consumer utility and subject to budget constraints. For simplicity, this study discusses only the demand function. The final goods of the three countries are heterogeneous goods, and thus their demand functions are assumed to be equations (9), (10), (11).

(9) $$q_{1}=\alpha -\beta p_{1}+\gamma p_{2}+\gamma p_{3}$$

(10) $$q_{2}=\alpha -\beta p_{2}+\gamma p_{3}+\gamma p_{1}$$

(11) $$q_{3}=\alpha -\beta p_{3}+\gamma p_{1}+\gamma p_{2}$$

where α,β,γ≥0. Parameter β is also called the self-price coefficient of demand, and the parameter γ is also known as the cross-price coefficient of demand. The relationship between the parameters β and γ denotes the degree of substitution between the final goods of the three countries. If γ=0, the three goods are independent with the highest heterogeneity. On the other hand, if γ=β, there is complete substitution between the three goods. The following proof will ensure the existence of this market structure, where the price of intermediate goods can be greater than zero, and the price of final goods is greater than zero.

This research presents a three-stage game using a dynamically complete information model. A subgame perfect equilibrium can be obtained by backward induction to identify an optimal trade policy for the two governments. The prices of final goods are equations (12), (13), (14).

(12) $$p_{1}=\frac{\left(2\beta +\gamma \right)\left(\alpha +\beta a_{1}+\beta a_{2}+\beta a_{3}\right)+\beta \left[\left(2\beta -\gamma \right)s_{1}+\gamma s_{2}+\gamma s_{3}+\left(2\beta -\gamma \right)t_{12}+{\gamma t}_{21}\right]}{2\left(\beta -\gamma \right)\left(2\beta +\gamma \right)}$$

(13) $$p_{2}=\frac{\left(2\beta +\gamma \right)\left(\alpha +\beta a_{1}+\beta a_{2}+\beta a_{3}\right)+\beta \left[\left(2\beta -\gamma \right)s_{2}+\gamma s_{3}+\gamma s_{1}+\left(2\beta -\gamma \right)t_{21}+{\gamma t}_{12}\right]}{2\left(\beta -\gamma \right)\left(2\beta +\gamma \right)}$$

(14) $$p_{3}=\frac{\left(2\beta +\gamma \right)\left(\alpha +\beta a_{1}+\beta a_{2}+\beta a_{3}\right)+\beta \left[\left(2\beta -\gamma \right)s_{3}+\gamma s_{1}+\gamma s_{2}+\gamma t_{12}+\gamma t_{21})\right]}{2\left(\beta -\gamma \right)\left(2\beta +\gamma \right)}$$

In the second stage, the intermediate goods firm in country i determines the intermediate goods price to maximize its respective profit, $\Omega_{i}$. The intermediate goods price can be obtained as a function of the tax rate by combining the first-order necessary conditions of the three firms (i.e., equation (15)):

(15) $$a_{i}=-\frac{-3\alpha +\left(\beta -2\gamma \right)\left(s_{1}+s_{2}+s_{3}+t_{12}+t_{21}\right)}{12\left(\beta -2\gamma \right)}$$

In the governments of the first stage, the three countries adopt an optimal trade policy for the export of final goods and determine whether a tax (positive value) or free trade (zero value) should be introduced to maximize welfare, $W_{i}$. The government of country 1 formulates an optimal tariff policy for the import of intermediate goods from country 2 and decides if a tariff (positive value) or a subsidy (negative value) should be implemented to maximize welfare, $W_{1}$. The government of country 2 creates an optimal tariff policy for the import of intermediate goods from country 1 and chooses between a tariff (positive value) and a subsidy (negative value) to maximize welfare, $W_{2}$. The welfare maximization strategies for the three countries are, respectively, $\max_{t_{12},s_{1}}W_{1}=T_{1}+\Omega_{1}+\Pi_{1}$, $\max_{t_{21},s_{2}}W_{2}=T_{2}+\Omega_{2}+\Pi_{2}$, and $\max_{s_{3}}W_{3}=T_{3}+\Omega_{3}+\Pi_{3}$.

This study explores the potential for a trade war by examining the optimal solutions across three scenarios: (1) $\left(1+\sqrt{145}\right)\gamma /12<\beta <\left(-1+\sqrt{33}\right)\gamma /4$; (2) $\beta >\left(-1+\sqrt{33}\right)\gamma /4$ or $\beta <\left(3+\sqrt{1945}\right)\gamma /44$; (3) $\left(3+\sqrt{1945}\right)\gamma /44<\beta <\left(1+\sqrt{145}\right)\gamma /12$, and the equilibrium analysis.

### **2.1** Relationship between the self-price coefficient of demand and the cross-price coefficient of demand: $\left(1+\sqrt{145}\right)\gamma /12<\beta <\left(-1+\sqrt{33}\right)\gamma /4$

Using the five first-order conditions for the three countries, optimal trade policies can be solved as follows.

(16) $$s_{1}^{*}+t_{12}^{*}=-\frac{\alpha \left(2\beta^{2}+\beta \gamma -4\gamma^{2}\right)}{22\beta^{3}-25\beta^{2}\gamma -14\beta \gamma^{2}+16\gamma^{3}}\;>0$$

(17) $$s_{2}^{*}+t_{21}^{*}=-\frac{\alpha \left(2\beta^{2}+\beta \gamma -4\gamma^{2}\right)}{22\beta^{3}-25\beta^{2}\gamma -14\beta \gamma^{2}+16\gamma^{3}}\;>0$$

(18) $$s_{3}^{*}=-\frac{\alpha \left(2\beta^{2}+\beta \gamma -4\gamma^{2}\right)}{22\beta^{3}-25\beta^{2}\gamma -14\beta \gamma^{2}+16\gamma^{3}}\;>0$$

The five first-order conditions are consolidated to derive three optimal conditional expressions. In particular, two of these five first-order conditions demonstrate collinearity or linear coincidence.

This study further investigates the trade-war model. Initially, in the first stage, the tariffs imposed by country 1, $t_{12}$, and country 2, $t_{21}$, become exogenous variables within the trade war scenario, allowing these two countries to manipulate $t_{12}$ and $t_{21}$ as desired. During this stage, the governments of the three countries establish an optimal export trade policy for the final goods, choosing between implementing a tax (positive value) and implementing free trade (zero value) to maximize welfare, $W_{i}$. The derivation of an optimal trade policy is based on three first-order conditions (i.e., Eqs. (16), (17), (18)). Accordingly, this study presents the following proposition.

Proposition 1 When the relationship between the self-price coefficient of demand and the cross-price coefficient of demand is $\left(1+\sqrt{145}\right)\gamma /12<\beta <\left(-1+\sqrt{33}\right)\gamma /4$, the trade war is likely to occur. Any reduction in the export tax on final goods will further exacerbate the trade war. In other words, a lower $s_{1}^{*}$ ($s_{2}^{*}$) will lead to a larger $t_{12}^{*}$ ($t_{21}^{*}$).

Proof: See the appendix.

According to Proposition 1, the trend of contemporary international division of labor is more significant. Moreover, a trade war between the two countries may occur, although not necessarily absolute, when the self-price coefficient of demand is neither significantly large nor significantly small relative to the cross-price coefficient of demand. At this stage, the import tariff policy for intermediate goods can be arbitrarily manipulated. However, such a trade war will also affect the government’s export trade policy for final goods. That is, the need to forgo an export policy for final goods offers stronger support for the trade war.

### 2.2 Relationship between the self-price coefficient of demand and the cross-price coefficient of demand: $\beta >\left(-1+\sqrt{33}\right)\gamma /4$ or $\beta <\left(3+\sqrt{1945}\right)\gamma /44$

Using the five first-order conditions for the three countries, optimal trade policies can be solved as follows.

(19) $$s_{1}^{*}+t_{12}^{*}=-\frac{3\alpha \left(2\beta +\gamma \right)\left(2\beta^{2}+\beta \gamma -4\gamma^{2}\right)}{2\left(76\beta^{4}-56\beta^{3}\gamma -87\beta^{2}\gamma^{2}+44\beta \gamma^{3}+20\gamma^{4}\right)}\;<0$$

(20) $$s_{2}^{*}+t_{21}^{*}=-\frac{3\alpha \left(2\beta +\gamma \right)\left(2\beta^{2}+\beta \gamma -4\gamma^{2}\right)}{2\left(76\beta^{4}-56\beta^{3}\gamma -87\beta^{2}\gamma^{2}+44\beta \gamma^{3}+20\gamma^{4}\right)}\;<0$$

(21) $$s_{3}^{*}=0$$

In the first stage, the tariffs imposed by country 1, $t_{12}$, and country 2, $t_{21}$, become exogenous variables within the trade war scenario, allowing these two countries to manipulate $t_{12}$ and $t_{21}$ as desired. At this stage, the governments of the three countries determine the optimal export policy for final goods, choosing between implementing a tax (positive value) and implementing free trade (zero value) to maximize welfare, $W_{i}$. The derivation of an optimal trade policy is based on three first-order conditions (i.e., Eqs. (19), (20), (21)). Accordingly, this study presents the following proposition.

Proposition 2 When the relationship between the self-price coefficient of demand and the cross-price coefficient of demand is $\beta >\left(-1+\sqrt{33}\right)\gamma /4$ or $\beta <\left(3+\sqrt{1945}\right)\gamma /44$, the trade war will not occur. Instead, both countries will subsidize each other's imports, fostering a more amicable trade relationship. A higher export tax on final goods will result in stronger reciprocity between country 1 and country 2. In essence, a higher $s_{1}^{*}$ ($s_{2}^{*}$) will lead to a lower $t_{12}^{*}$ ($t_{21}^{*}$).

Proof: See the appendix.

According to Proposition 2, even with the prevalence of international division of labor, the realization of a trade war might not materialize when the self-price coefficient of demand is sufficiently large or small relative to the cross-price coefficient of demand. On the contrary, country 1 and country 2 may opt for a mutually beneficial import subsidy. This outcome stems from the monopolistic power wielded by intermediary goods firms due to the international division of labor. This monopoly could lead to the transfer of tariffs imposed on the export of intermediary goods to the country's final goods, lowering the competitiveness of the final goods. Consequently, the tendency to impose tariffs could decline, while the tendency to adopt import subsidies could increase to bolster the competitiveness of final goods exports.

### 2.3 Relationship between the self-price coefficient of demand and the cross-price coefficient of demand**:**$\left(3+\sqrt{1945}\right)\gamma /44<\beta <\left(1+\sqrt{145}\right)\gamma /12$

Using the five first-order conditions for the three countries, optimal trade policies can be solved as follows.

(22) $$s_{1}^{*}+t_{12}^{*}=-\frac{3\alpha \left(2\beta +\gamma \right)\left(2\beta^{2}+\beta \gamma -4\gamma^{2}\right)}{2\left(76\beta^{4}-56\beta^{3}\gamma -87\beta^{2}\gamma^{2}+44\beta \gamma^{3}+20\gamma^{4}\right)}>0$$

(23) $$s_{2}^{*}+t_{21}^{*}=-\frac{3\alpha \left(2\beta +\gamma \right)\left(2\beta^{2}+\beta \gamma -4\gamma^{2}\right)}{2\left(76\beta^{4}-56\beta^{3}\gamma -87\beta^{2}\gamma^{2}+44\beta \gamma^{3}+20\gamma^{4}\right)}>0$$

(24) $$s_{3}^{*}=0$$

In the first stage of the trade-war model, the tariff of country 1, $t_{12}$, and the tariff of country 2, $t_{21}$, become exogenous variables. That is, both countries can manipulate $t_{12}$ and $t_{21}$ at desired. At this stage, the governments of the three countries determine the optimal export policy for final goods and decide between imposing a tax (positive value) or adopting free trade (zero value) to maximize welfare, $W_{i}$. An optimal trade policy can be obtained from the three first-order conditions for the firms of the three countries (that is, Eqs. (22), (23), (24)). Accordingly, this study presents the following proposition.

Proposition 3 When the relationship between the self-price coefficient of demand and the cross-price coefficient of demand is $\left(3+\sqrt{1945}\right)\gamma /44<\beta <\left(1+\sqrt{145}\right)\gamma /12$, the trade war is likely to occur. Any decrease in the export tax on final goods will further exacerbate the trade war. In essence, a reduction $s_{1}^{*}$ ($s_{2}^{*}$) will result in an increase in $t_{12}^{*}$ ($t_{21}^{*}$).

Proof: See the appendix.

According to Proposition 1, when $\left(1+\sqrt{145}\right)\gamma /12<$ $\beta <\left(-1+\sqrt{33}\right)\gamma /4$, there’s a potential for a trade war. Proposition 3 suggests that a trade war might also occur when $\left(3+\sqrt{1945}\right)\gamma /44<\beta <\left(1+\sqrt{145}\right)\gamma /12$. Combining these propositions, a trade war might arise when $1.0705\gamma =\left(3+\sqrt{1945}\right)\gamma /44<\beta <\left(-1+\sqrt{33}\right)\gamma /4=1.18614\gamma$. In essence, this indicates that a trade war might occur when the self-price coefficient of demand is neither significantly large nor significantly small relative to the cross-price coefficient of demand. During this phase, the import tariff policy for intermediate goods can be arbitrarily manipulated. However, such a trade war will also impact the government’s export trade policy for final goods, indicating that forgoing the final product export policy will provide stronger support for the occurrence of a trade war.

### 2.4 Equilibrium analysis

In addition to exploring the trade relationship, this study conducts an equilibrium analysis of $\left(1+\sqrt{145}\right)\gamma /12<\beta <\left(-1+\sqrt{33}\right)\gamma /4$ and $\beta >\left(-1+\sqrt{33}\right)\gamma /4$ or $\beta <\left(1+\sqrt{145}\right)\gamma /12$. More specifically, it determines the social welfare, total taxes, profits of intermediate goods and final goods firms, outputs, and prices of final and intermediate goods in equilibrium.

First, when $\left(1+\sqrt{145}\right)\gamma /12<\beta <\left(-1+\sqrt{33}\right)\gamma /4$, the following equilibrium can be obtained by incorporating equations (16), (17), (18) into equations (1), (2), (3), (4), (5), (6), (7), (8), (9), (10), (11), (12), (13), (14), (15):

(25) $$W_{i}^{*}=\frac{\alpha^{2}\beta \left(228\beta^{5}-332\beta^{4}\gamma -311\beta^{3}\gamma^{2}+508\beta^{2}\gamma^{3}+100\beta \gamma^{4}-192\gamma^{5}\right)}{4\left(\beta -2\gamma \right){\left(22\beta^{3}-25\beta^{2}\gamma -14\beta \gamma^{2}+16\gamma^{3}\right)}^{2}}$$

(26) $$T_{i}^{*}=-\frac{\alpha^{2}\beta \left(6\beta^{2}-\beta \gamma -6\gamma^{2}\right)\left(2\beta^{2}+\beta \gamma -4\gamma^{2}\right)}{2{\left(22\beta^{3}-25\beta^{2}\gamma -14\beta \gamma^{2}+16\gamma^{3}\right)}^{2}}$$

(27) $$\Omega_{i}^{*}=\frac{3\alpha^{2}\beta \left(\beta -\gamma \right){\left(-6\beta^{2}+\beta \gamma +6\gamma^{2}\right)}^{2}}{2\left(\beta -2\gamma \right){\left(22\beta^{3}-25\beta^{2}\gamma -14\beta \gamma^{2}+16\gamma^{3}\right)}^{2}}$$

(28) $$\Pi_{i}^{*}=\frac{\alpha^{2}\beta {\left(6\beta^{2}-\beta \gamma -6\gamma^{2}\right)}^{2}}{4{\left(22\beta^{3}-25\beta^{2}\gamma -14\beta \gamma^{2}+16\gamma^{3}\right)}^{2}}$$

(29) $$q_{i}^{*}=\frac{\alpha \beta \left(6\beta^{2}-\beta \gamma -6\gamma^{2}\right)}{2\left(22\beta^{3}-25\beta^{2}\gamma -14\beta \gamma^{2}+16\gamma^{3}\right)}$$

(30) $$p_{i}^{*}=\frac{\alpha \left(38\beta^{3}-49\beta^{2}\gamma -22\beta \gamma^{2}+32\gamma^{3}\right)}{2\left(\beta -2\gamma \right)\left(22\beta^{3}-25\beta^{2}\gamma -14\beta \gamma^{2}+16\gamma^{3}\right)}$$

(31) $$a_{i}^{*}=\frac{\alpha \left(6\beta^{3}-7\beta^{2}\gamma -5\beta \gamma^{2}+6\gamma^{3}\right)}{\left(\beta -2\gamma \right)\left(22\beta^{3}-25\beta^{2}\gamma -14\beta \gamma^{2}+16\gamma^{3}\right)}$$

Second, when $\beta >\left(-1+\sqrt{33}\right)\gamma /4$ or $\beta <\left(1+\sqrt{145}\right)\gamma /12$, the following equilibrium can be obtained by introducing Eqs. (19), (20), (21) and Eqs. (22), (23), (24) in Eqs. (1), (2), (3), (4), (5), (6), (7), (8), (9), (10), (11), (12), (13), (14), (15).

$$W_{1}^{*}=W_{2}^{*}=$$

(32) $$\frac{\alpha^{2}\beta \left(10480\beta^{7}-6592\beta^{6}\gamma -25104\beta^{5}\gamma^{2}+9960\beta^{4}\gamma^{3}+20601\beta^{3}\gamma^{4}-2388\beta^{2}\gamma^{5}-5788\beta \gamma^{6}-1088\gamma^{7}\right)}{16\left(\beta -2\gamma \right){\left(76\beta^{4}-56\beta^{3}\gamma -87\beta^{2}\gamma^{2}+44\beta \gamma^{3}+20\gamma^{4}\right)}^{2}}$$

$$W_{3}^{*}=$$

(33) $$\frac{\alpha^{2}\beta \left(10624\beta^{7}-6976\beta^{6}\gamma -25476\beta^{5}\gamma^{2}+11388\beta^{4}\gamma^{3}+20391\beta^{3}\gamma^{4}-3300\beta^{2}\gamma^{5}-5476\beta \gamma^{6}-1184\gamma^{7}\right)}{16\left(\beta -2\gamma \right){\left(76\beta^{4}-56\beta^{3}\gamma -87\beta^{2}\gamma^{2}+44\beta \gamma^{3}+20\gamma^{4}\right)}^{2}}$$

(34) $$T_{1}^{*}=T_{2}^{*}=-\frac{3\alpha^{2}\beta {\left(2\beta +\gamma \right)}^{2}\left(22\beta^{2}-3\beta \gamma -22\gamma^{2}\right)\left(2\beta^{2}+\beta \gamma -4\gamma^{2}\right)}{8{\left(76\beta^{4}-56\beta^{3}\gamma -87\beta^{2}\gamma^{2}+44\beta \gamma^{3}+20\gamma^{4}\right)}^{2}}$$

(35) $$T_{3}^{*}=0$$

(36) $$\Omega_{i}^{*}=\frac{3\alpha^{2}\beta \left(\beta -\gamma \right){\left(40\beta^{3}+10\beta^{2}\gamma -41\beta \gamma^{2}-14\gamma^{3}\right)}^{2}}{8\left(\beta -2\gamma \right){\left(76\beta^{4}-56\beta^{3}\gamma -87\beta^{2}\gamma^{2}+44\beta \gamma^{3}+20\gamma^{4}\right)}^{2}}$$

(37) $$\Pi_{1}^{*}=\Pi_{2}^{*}=\frac{\alpha^{2}\beta {\left(2\beta +\gamma \right)}^{2}{\left(22\beta^{2}-3\beta \gamma -22\gamma^{2}\right)}^{2}}{16{\left(76\beta^{4}-56\beta^{3}\gamma -87\beta^{2}\gamma^{2}+44\beta \gamma^{3}+20\gamma^{4}\right)}^{2}}$$

(38) $$\Pi_{3}^{*}=\frac{\alpha^{2}\beta {\left(32\beta^{3}-2\beta^{2}\gamma -29\beta \gamma^{2}+2\gamma^{3}\right)}^{2}}{16{\left(76\beta^{4}-56\beta^{3}\gamma -87\beta^{2}\gamma^{2}+44\beta \gamma^{3}+20\gamma^{4}\right)}^{2}}$$

(39) $$q_{1}^{*}=q_{2}^{*}=\frac{\alpha \beta \left(2\beta +\gamma \right)\left(22\beta^{2}-3\beta \gamma -22\gamma^{2}\right)}{4\left(76\beta^{4}-56\beta^{3}\gamma -87\beta^{2}\gamma^{2}+44\beta \gamma^{3}+20\gamma^{4}\right)}$$

(40) $$q_{3}^{*}=\frac{\alpha \beta \left(32\beta^{3}-2\beta^{2}\gamma -29\beta \gamma^{2}+2\gamma^{3}\right)}{4\left(76\beta^{4}-56\beta^{3}\gamma -87\beta^{2}\gamma^{2}+44\beta \gamma^{3}+20\gamma^{4}\right)}$$

(41) $$p_{1}^{*}=p_{2}^{*}=\frac{\alpha \left(10\beta^{2}-3\beta \gamma -10\gamma^{2}\right)\left(26\beta^{2}-15\beta \gamma -8\gamma^{2}\right)}{4\left(\beta -2\gamma \right)\left(76\beta^{4}-56\beta^{3}\gamma -87\beta^{2}\gamma^{2}+44\beta \gamma^{3}+20\gamma^{4}\right)}$$

(42) $$p_{3}^{*}=\frac{\alpha \left(272\beta^{4}-246\beta^{3}\gamma -331\beta^{2}\gamma^{2}+222\beta \gamma^{3}+80\gamma^{4}\right)}{4\left(\beta -2\gamma \right)\left(76\beta^{4}-56\beta^{3}\gamma -87\beta^{2}\gamma^{2}+44\beta \gamma^{3}+20\gamma^{4}\right)}$$

(43) $$a_{i}^{*}=\frac{\alpha \left(\beta -\gamma \right)\left(40\beta^{3}+10\beta^{2}\gamma -41\beta \gamma^{2}-14\gamma^{3}\right)}{2\left(\beta -2\gamma \right)\left(76\beta^{4}-56\beta^{3}\gamma -87\beta^{2}\gamma^{2}+44\beta \gamma^{3}+20\gamma^{4}\right)}$$

Equations (25), (26), (27), (28), (29), (30), (31), (32), (33), (34), (35), (36), (37), (38), (39), (40), (41), (42), (43) demonstrate that the equilibrium results have no variable that contributes to the trade war or subsidies, regardless of whether the trade war emerges under $\left(3+\sqrt{1945}\right)\gamma /44<\beta <\left(-1+\sqrt{33}\right)\gamma /4$ or if trade subsidies result from $\beta >\left(-1+\sqrt{33}\right)\gamma /4$ or $\beta <\left(3+\sqrt{1945}\right)\gamma /44$. Accordingly, Proposition 4 is derived.

Proposition 4 Regardless of whether the trade war is caused by $\left(3+\sqrt{1945}\right)\gamma /44<\beta <\left(-1+\sqrt{33}\right)\gamma /4$ or trade subsidies resulting from $\beta >\left(-1+\sqrt{33}\right)\gamma /4$ or $\beta <\left(3+\sqrt{1945}\right)\gamma /44$, the equilibrium solutions for the production output of the three countries $q_{i}^{*}$, final goods price $p_{i}^{*}$, profit of final goods firm $\Pi_{i}^{*}$, intermediate goods price $a_{i}^{*}$, profit of intermediate goods firm $\Omega_{i}^{*}$, government tax $T_{i}^{*}$, and social welfare $W_{i}^{*}$ remain unaffected by the scale of the trade war or subsidy.

Proof: See the appendix.

From Proposition 4, the conclusion can be drawn that the scale of the trade war or the trade subsidy does not affect the equilibrium variables. Factors such as upstream and downstream firms, government taxation, and social welfare remain unaffected by the tariff or subsidy, implying that the trade war or subsidy may be declarative. The above results can also be verified by Ahmad and Ahmad (2023) [1]. They also found from empirical research that in the US trade war, high tariffs are not completely passed on to consumers for intermediate goods such as steel products.

Therefore, for the trade war or trade subsidy to have a substantial effect, countries 1 and 2 must jointly or unilaterally alter the market structure of the international division of labor. This change should disrupt the complementary relationship among intermediate goods, prompting the outsourcing or OEM industries to return to their respective domestic countries. Consequently, the significant contributions of the findings of this study to trade policy, export policy, and the overall economy can be summarized as follows. In the prevailing scenario of international trade with the international division of labor, trade wars are merely symbolic declarations without influencing the variables of international trade. Although a high-tariff trade war on intermediate goods may seem to impact the export of intermediate goods from the opponent country, it will increase the production costs of final goods in the home country. The two countries compete in the final product market; they balance gains and losses, resulting in a net effect of zero from the trade war. Hence, this study's crucial insight lies in the discovery that while the international division of labor fosters cooperative effects, trade wars remain ineffectual. To render the trade war effective, it becomes imperative to abandon the international division of labor and opt for horizontal integration, repatriating the industries of the international division of labor to domestic production. Only after restructuring the industrial landscape in this manner can the trade war wield any efficacy.

The aforementioned results of this study tend to be consistent with the conclusion of Toraubally (2022) [43], suggesting that the traditional Ricardo-Haberlerian (1817; 1936) [[22], [36]] theory of comparative advantage and the game of trade war will lead to nonco-existence. Furthermore, our research conclusions are also consistent with the empirical research of Gur and Dilek (2023) [21]. They suggested that the United States cannot fully consolidate its technological advantages over China through the trade war alone. In addition to promoting the return of industries, the United States should also create an integrated policy framework.

The trade wars that began in 2014 include conflicts between the United States and China, Japan and South Korea, and the United States and Russia. To make these trade wars effective, these countries still need to adjust the industrial structure of the international division of labor with respect to intermediate goods.

Although the development of the topic of this research can be considered subjective perception, according to the definition of Professor M. Friedman of the University of Chicago, the quality of an economic model does not depend on whether the model is consistent with reality, but rather on its predictive and explanatory abilities. Therefore, although the results of this study are based on an abstract theoretical model, it does not impede its economic explanatory and predictive capacity. In other words, while this study may have fewer empirical cases, its findings can be generalized and applied to various scenarios of trade wars in the future. But without loss of generality, the findings of this study can be applied to more cases of trade wars in the future. The study’s conclusions suggest the potential to establish more empirical models in subsequent research to validate its practical application, which requires further verification in follow-up studies.

The interpretation of the results in this study is based on the theoretical model, so it is the result after abstraction. To apply these findings in practical scenarios without bias, future empirical research is recommended. This approach would lend practical value to these mathematical equations. Essentially, estimating these parameters remains theoretical, possibly leading to discrepancies between the theoretical and actual variables. However, optimizing the model is unlikely to cause significant alterations. Therefore, the model in this study provides directional insights and has a reference value for policy making. Subsequent research directions could involve testing its sensitivity through empirical studies.

To determine the stability and consistency of the overall model, especially in the evaluation and selection of multiple parameters and variables for real-world applications, a sensitivity analysis is conducted to validate the research results. This is especially crucial if there are variations in the causal relationships within these models. Such an analysis helps in effectively managing risks.

Typically, in empirical model sensitivity analysis, if the original research results exhibit stability, the introduction of one or more variables will lead to changes in the magnitude of the effects, but the direction of the impact of the independent variables on the dependent variable will remain unchanged. Similarly, in the sensitivity analysis of theoretical models, if the original research results exhibit stability when the model structure is altered, the equilibrium solutions will change. However, the direction of correlation among equilibrium variables will still follow specific patterns. The sensitivity analysis will be conducted below by altering the structure of the theoretical model.

Firstly, the original international division of labor among three countries in intermediate goods will be changed to an international division of labor among two countries in intermediate goods. Under the assumption that all other conditions remain unchanged, we exclude the third country. Through sensitivity analysis, the model is analyzed using subgame perfect equilibrium, and we obtain correlations among the variables similar to those in equations (16), (17), (18), (19), (20), (21), (22), (23), (24). These correlations continue to exhibit the same regular patterns, and the results are presented as follows (i.e., equations (44), (45)).

(44) $$s_{1}^{*}+t_{12}^{*}=-\frac{\alpha \left(4\beta^{2}+2\beta \gamma -3\gamma^{2}\right)}{20\beta^{3}-4\beta^{2}\gamma -10\beta \gamma^{2}+3\gamma^{3}}$$

(45) $$s_{2}^{*}+t_{21}^{*}=-\frac{\alpha \left(4\beta^{2}+2\beta \gamma -3\gamma^{2}\right)}{20\beta^{3}-4\beta^{2}\gamma -10\beta \gamma^{2}+3\gamma^{3}}$$

Through subsequent equilibrium analysis, similar inferences can be obtained as those in Propositions 1, 2, and 3. More importantly, the subsequent analysis leads to the same conclusion as that presented in Proposition 4. Therefore, the sensitivity analysis reveals that if two countries engage in an international division of labor, it provides support in the same direction as the conclusions drawn in this study.

Secondly, the original international division of labor among three countries in intermediate goods is expanded to an international division of labor among four countries in intermediate goods. In other words, under the assumption that all other conditions remain unchanged, a fourth country is introduced to participate in the international division of labor. Through sensitivity analysis, the model is analyzed using subgame perfect equilibrium, and we obtain correlations among the variables similar to those in equations (16), (17), (18), (19), (20), (21), (22), (23), (24). These correlations continue to exhibit the same regular patterns, and the results are presented below (i.e., equations (46), (47), (48), (49)).

(46) $$s_{1}^{*}+t_{12}^{*}=-\frac{\alpha \left(4\beta^{2}+2\beta \gamma -15\gamma^{2}\right)}{76\beta^{3}-160\beta^{2}\gamma -24\beta \gamma^{2}+135\gamma^{3}}$$

(47) $$s_{2}^{*}+t_{21}^{*}=-\frac{\alpha \left(4\beta^{2}+2\beta \gamma -15\gamma^{2}\right)}{76\beta^{3}-160\beta^{2}\gamma -24\beta \gamma^{2}+135\gamma^{3}}$$

(48) $$s_{3}^{*}=-\frac{\alpha \left(4\beta^{2}+2\beta \gamma -15\gamma^{2}\right)}{76\beta^{3}-160\beta^{2}\gamma -24\beta \gamma^{2}+135\gamma^{3}}$$

(49) $$s_{4}^{*}=-\frac{\alpha \left(4\beta^{2}+2\beta \gamma -15\gamma^{2}\right)}{76\beta^{3}-160\beta^{2}\gamma -24\beta \gamma^{2}+135\gamma^{3}}$$

Through subsequent equilibrium analysis, similar inferences can be drawn as derived in Proposition 1, Proposition 2, and Proposition 3. More importantly, the subsequent analysis reaches the same conclusion as that in Proposition 4. Therefore, the sensitivity analysis reveals that when four countries participate in an international division of labor, it aligns with the findings of this study.

Based on the sensitivity analyses of the two scenarios mentioned above, the research findings remain consistent in all three cases. Therefore, by sensitivity analysis, this study confirms the stability and consistency of the research results.

Thirdly, in Fig. 1, it is evident that this model is divided into three stages, which form a dynamic three-stage model. In the initial stage, the governments of the three producing countries intervene in the export of final goods of each country and may adopt measures such as export taxation, free trade, or export subsidies. However, only in this initial stage, it is assumed that the trade policies of the three countries’ governments are decided simultaneously, which means a static model analysis in this first stage.

To examine dynamic analysis in addressing the complexities of the trade war, the following sensitivity analysis will transition the static analysis of the first stage of the game into a dynamic approach. Employing dynamic methods to analyze complex trade wars can enhance practical applicability and increase the reference value for real-world applications.

In the following games, the first stage of this model will be transformed from a static form to a dynamic one. Without loss of generality, assume that in the first stage, the government of country 1 first determines the trade policies $s_{1}$ and $t_{12}$, and then the governments of country 2 and country 3 decide the trade policies $s_{2}$, $t_{21}$, and $s_{3}$ respectively.

Using sensitivity analysis, the model is evaluated using subgame perfect equilibrium. In the first stage, the reaction functions of $s_{2}$, $t_{21}$, and $s_{3}$ are derived through backward induction, followed by obtaining the equilibrium solutions of $s_{1}$ and $t_{12}$ by employing backward induction to the earlier stage. These optimal solutions are then reintegrated into the reaction function of each stage and correlations among variables similar to those of equations (16), (17), (18), (19), (20), (21), (22), (23), (24) are obtained. These correlations persist in demonstrating the same consistent patterns, and the results are presented in the following (i.e., equations (50), (51), (52)).

(50) $$s_{1}^{*}+t_{12}^{*}=\frac{\alpha \left(-2304\beta^{7}+64\beta^{6}\gamma +7636\beta^{5}\gamma^{2}-412\beta^{4}\gamma^{3}-7787\beta^{3}\gamma^{4}+200\beta^{2}\gamma^{5}+2500\beta \gamma^{6}+112\gamma^{7}\right)}{19264\beta^{8}-23104\beta^{7}\gamma -44412\beta^{6}\gamma^{2}+52284\beta^{5}\gamma^{3}+34869\beta^{4}\gamma^{4}-38574\beta^{3}\gamma^{5}-10372\beta^{2}\gamma^{6}+9304\beta \gamma^{7}+768\gamma^{8}}$$

(51) $$s_{2}^{*}+t_{21}^{*}=\frac{\alpha \left(-1824\beta^{7}-608\beta^{6}\gamma +6676\beta^{5}\gamma^{2}+1004\beta^{4}\gamma^{3}-7193\beta^{3}\gamma^{4}-640\beta^{2}\gamma^{5}+2332\beta \gamma^{6}+208\gamma^{7}\right)}{19264\beta^{8}-23104\beta^{7}\gamma -44412\beta^{6}\gamma^{2}+52284\beta^{5}\gamma^{3}+34869\beta^{4}\gamma^{4}-38574\beta^{3}\gamma^{5}-10372\beta^{2}\gamma^{6}+9304\beta \gamma^{7}+768\gamma^{8}}$$

$$s_{3}^{*}=$$

(52) $$\frac{\alpha \left(-1824\beta^{7}-608\beta^{6}\gamma +6676\beta^{5}\gamma^{2}+1004\beta^{4}\gamma^{3}-7193\beta^{3}\gamma^{4}-640\beta^{2}\gamma^{5}+2332\beta \gamma^{6}+208\gamma^{7}\right)}{19264\beta^{8}-23104\beta^{7}\gamma -44412\beta^{6}\gamma^{2}+52284\beta^{5}\gamma^{3}+34869\beta^{4}\gamma^{4}-38574\beta^{3}\gamma^{5}-10372\beta^{2}\gamma^{6}+9304\beta \gamma^{7}+768\gamma^{8}}$$

Through subsequent equilibrium analysis, similar inferences are drawn to Proposition 1, Proposition 2, and Proposition 3. More crucially, the subsequent analysis arrives at the same conclusion as expressed in Proposition 4. Therefore, this sensitivity analysis indicates that if two countries participate in an international division of labor, it reinforces the same trend as the findings of this study.

Using the sensitivity analysis of the third scenario mentioned above, the research findings align with the original model. Therefore, by sensitivity analysis, this study confirms the stability and consistency of these research results.

## 3 Conclusions

The trade war emerged due to arbitrarily high tariffs imposed by Country 1 and Country 2 on imports from each other as a punitive measure for unfair trade practices. However, the existing literature lacks comprehensive theoretical models on the trade war. Thus, this study aims to establish a model that increases theoretical comprehension of trade wars. This theoretical model focusses on the upstream industries in three countries, where their intermediate goods show complementarity to each other. Furthermore, downstream industry goods are exported to a competitive market in a fourth country. The model elucidates the essential conditions dictating the occurrence of a trade war and those conducive to the emergence of trade subsidies. Additionally, we conducted tests to evaluate the circumstances under which a trade war or a subsidy can be effective.

This study reveals three main findings. First, when the self-price coefficient falls below 1.18614 times the cross-price coefficient and exceeds 1.0705 times the cross-price coefficient, the likelihood of a trade war emerges, primarily to protect domestic final goods firms. A reduced export tax on final goods tends to escalate the intensity of the trade war. Second, if the self-price coefficient exceeds 1.18614 times the cross-price coefficient or descends below 1.0705 times the cross-price coefficient, a trade subsidy is likely to be implemented. This results in reciprocal actions between the two countries but a higher tax burden on domestic firms producing final goods in both countries. Finally, regardless of a trade war or trade subsidy, or their magnitude, the scale of the seven equilibrium variables encompassing taxes, prices, quantities, profits, and social welfare remains unaffected.

Hence, for the trade war or trade subsidy to yield results, both nations must reform their existing market structure, pivoting from the international division of labor towards domesticating the industry through horizontal integration. A trade war escalates hostilities, whereas a trade subsidy fosters amicable trade relations.

The limitation of this study lies in its inherent complexity due to the multifaceted nature of factors within the trade-war scenario. The intricate nature of this subject presents challenges in establishing a clear entry point, leading to a partial gap between the scope of the title and the actual discussion. Addressing these complexities requires imposing numerous research constraints, which can potentially hinder an entirely objective analysis. In essence, this study remains purely theoretical as it abstracts real-world scenarios into a model; consequently, the theoretical variables may not fully align with practical counterparts, representing a limitation.

Future research should aim to bridge this gap by translating theoretical variables into empirically verifiable factors and conducting empirical research to validate these models. The present study, being purely theoretical, lacks real-world data application for case studies and simulation analysis. Thus, future research should focus on improving the model to enhance its practical applicability by integrating real-world data.

## Ethics statement

Review and approval by an ethics committee were deemed unnecessary for this study since it involved theoretical analysis without the collection or analysis of new data. Informed consent was also unnecessary for the same reason.

## Data availability statement

No data was used for the research described in the article.

## Additional information

No additional information is available for this paper.

## CRediT authorship contribution statement

**Chun-Hung Chen:** Writing – review & editing, Writing – original draft, Visualization, Validation, Supervision, Software, Resources, Project administration, Methodology, Investigation, Formal analysis, Data curation, Conceptualization.

## Declaration of competing interest

The authors declare that they have no known competing financial interests or personal relationships that could have appeared to influence the work reported in this paper.

## Appendix

Proposition 1 When the relationship between the self-price coefficient of demand and the cross-price coefficient of demand is $\left(1+\sqrt{145}\right)\gamma /12<\beta <\left(-1+\sqrt{33}\right)\gamma /4$, the trade war is likely to occur. Any reduction in the export tax on final goods will further exacerbate the trade war. In other words, a lower $s_{1}^{*}$ ($s_{2}^{*}$) will lead to a larger $t_{12}^{*}$ ($t_{21}^{*}$).

Proof.

(1) This item will prove that equations (12), (13), (14) are as follows. Substitute equations (9), (10), (11) into equations (6), (7), (8), respectively, to obtain $\pi_{1}=\left[\beta p_{1}-\alpha -\gamma \left(p_{2}+p_{3}\right)\right]\left(a_{1}+a_{2}+a_{3}-p_{1}+s_{1}+t_{12}\right)$, and $\pi_{2}=\left(\beta p_{2}-\alpha -\gamma \left(p_{1}+p_{3}\right)\right)\left(a_{1}+a_{2}+a_{3}-p_{2}+s_{2}+t_{21}\right)$, and $\pi_{3}=\left(\beta p_{3}-\alpha -\gamma \left(p_{1}+p_{2}\right)\right)\left(a_{1}+a_{2}+a_{3}-p_{3}+s_{3}\right)$. From the first-order necessary conditions $\partial \pi_{1}/\partial p_{1}=0$, and $\partial \pi_{2}/\partial p_{2}=0$, and $\partial \pi_{3}/\partial p_{3}=0$. When the above three equations are combined, equations (12), (13), (14) are obtained.
(2) This item will prove that equation (15) is as follows. Bring the results of equations (12), (13), (14) back to equations (9), (10), (11), and then substitute equations (9), (10), (11) back to equation (5), the three equations of $\Omega_{1}$, $\Omega_{2}$, and $\Omega_{3}$ are obtained respectively. $\Omega_{1}=\beta a_{1}\left[3\alpha -\left(\beta -2\gamma \right)\left(3a_{1}+3a_{2}+3a_{3}+s_{1}+s_{2}+s_{3}+t_{12}+t_{21}\right)\right]/\left[2\left(\beta -\gamma \right)\right]$, and $\Omega_{2}=\beta a_{2}\left[3\alpha -\left(\beta -2\gamma \right)\left(3a_{1}+3a_{2}+3a_{3}+s_{1}+s_{2}+s_{3}+t_{12}+t_{21}\right)\right]/\left[2\left(\beta -\gamma \right)\right]$, and $\Omega_{3}=\beta a_{3}[3\alpha -\left(\beta -2\gamma \right)\left(3a_{1}+3a_{2}+3a_{3}+s_{1}+s_{2}+s_{3}+t_{12}+t_{21}\right)/\left[2\left(\beta -\gamma \right)\right]$. From the first-order necessary conditions $\partial \Omega_{1}/\partial a_{1}=0$, and $\partial \Omega_{2}/\partial a_{2}=0$, and $\partial \Omega_{3}/\partial a_{3}=0$. When the above three equations are combined, the first equation (15) is obtained, which is the general equation of the three equations.
(3) This item will prove that equations (16), (17), (18) are as follows. Bring back the result of the general equation of equation (15) to equations (2), (3), (4), (5), (6), (7), (8), (9), (10), (11), (12), (13), (14), and these results are substituted back into the first equation (1), then the three equations $W_{1}$, $W_{2}$, and $W_{3}$ are obtained respectively. The contents of $W_{1}$, $W_{2}$, and $W_{3}$ are too long, so they will not be listed in detail here. From the first-order necessary conditions $\partial W_{1}/\partial s_{1}=0$, and $\partial W_{2}/\partial s_{2}=0$, and $\partial W_{3}/\partial s_{3}=0$, and $\partial W_{1}/\partial t_{12}=0$, and $\partial W_{2}/\partial t_{21}=0$. When the above five equations are combined, equations (16), (17), (18) are obtained. They are as follows (i.e., equations (53), (54), (55)):

(53) $$s_{1}^{*}+t_{12}^{*}=-\frac{\alpha \left(2\beta^{2}+\beta \gamma -4\gamma^{2}\right)}{22\beta^{3}-25\beta^{2}\gamma -14\beta \gamma^{2}+16\gamma^{3}}\text{,}$$

(54) $$s_{2}^{*}+t_{21}^{*}=-\frac{\alpha \left(2\beta^{2}+\beta \gamma -4\gamma^{2}\right)}{22\beta^{3}-25\beta^{2}\gamma -14\beta \gamma^{2}+16\gamma^{3}}\text{,}$$

(55) $$s_{3}^{*}=-\frac{\alpha \left(2\beta^{2}+\beta \gamma -4\gamma^{2}\right)}{22\beta^{3}-25\beta^{2}\gamma -14\beta \gamma^{2}+16\gamma^{3}}\text{.}$$

(4) This item will prove that the denominator $22\beta^{3}-25\beta^{2}\gamma -14\beta \gamma^{2}+16\gamma^{3}>0$ in equations (53), (54), (55). Bring the results obtained in equations (53), (54), (55) and the above three items back to equations (9), (10), (11), and the following equation (56) can be obtained.

$$q_{1}=q_{2}=q_{3}=\frac{\alpha \beta \left(6\beta^{2}-\beta \gamma -6\gamma^{2}\right)}{2\left(22\beta^{3}-25\beta^{2}\gamma -14\beta \gamma^{2}+16\gamma^{3}\right)}$$

(56) $$=\frac{6\alpha \beta \left[\beta +\left(-1+\sqrt{145}\right)\gamma /12\right]\left[\beta -\left(1+\sqrt{145}\right)\gamma /12\right]}{2\left(22\beta^{3}-25\beta^{2}\gamma -14\beta \gamma^{2}+16\gamma^{3}\right)}$$

Because $q_{1}=q_{2}=q_{3}>0$, and because $\beta +\left(-1+\sqrt{145}\right)\gamma /12>0$. When $\left(1+\sqrt{145}\right)\gamma /12<\beta$, then $22\beta^{3}-25\beta^{2}\gamma -14\beta \gamma^{2}+16\gamma^{3}>0$ in the denominator of the quantity can be obtained.

(5) This item will prove that equations (53), (54), (55) are all greater than 0. Rewrite equations (53), (54), (55) as equation (57).

$$s_{1}^{*}+t_{12}^{*}=s_{2}^{*}+t_{21}^{*}=s_{3}^{*}=-\frac{\alpha \left(2\beta^{2}+\beta \gamma -4\gamma^{2}\right)}{22\beta^{3}-25\beta^{2}\gamma -14\beta \gamma^{2}+16\gamma^{3}}$$

(57) $$=\frac{-2\alpha \left[\beta +\left(1+\sqrt{33}\right)\gamma /4\right]\left[\beta -\left(-1+\sqrt{33}\right)\gamma /4\right]}{22\beta^{3}-25\beta^{2}\gamma -14\beta \gamma^{2}+16\gamma^{3}}$$

It is known from the fourth item that $22\beta^{3}-25\beta^{2}\gamma -14\beta \gamma^{2}+16\gamma^{3}>0$, and because $\beta +\left(1+\sqrt{33}\right)\gamma /4>0$. When $\beta <\left(-1+\sqrt{33}\right)\gamma /4$, so $s_{1}^{*}+t_{12}^{*}=s_{2}^{*}+t_{21}^{*}=s_{3}^{*}>0$.

(6) This item will prove that when $\beta <\left(1+\sqrt{145}\right)\gamma /12$ or $\beta >\left(-1+\sqrt{33}\right)\gamma /4$ will cause a contradiction. First, if $\beta <\left(1+\sqrt{145}\right)\gamma /12$, since $q_{1}=q_{2}=q_{3}>0$, the denominator of quantity is $22\beta^{3}-25\beta^{2}\gamma -14\beta \gamma^{2}+16\gamma^{3}<0$, and because $\beta <\left(1+\sqrt{145}\right)\gamma /12=1.0868\gamma$, so $\beta <\left(-1+\sqrt{33}\right)\gamma /4=1.18614\gamma$. And $s_{3}^{*}<0$ is obtained from equation (45), therefore, the contradiction holds. Secondly, when $\beta >\left(-1+\sqrt{33}\right)\gamma /4$, since $q_{1}=q_{2}=q_{3}>0$, the denominator of quantity is $22\beta^{3}-25\beta^{2}\gamma -14\beta \gamma^{2}+16\gamma^{3}>0$, and because $\beta >\left(-1+\sqrt{33}\right)\gamma /4$. And $s_{3}^{*}<0$ is obtained from equation (54), so the contradiction holds.

(7) This item will prove that a trade war may occur in equation (54), and the lower the final goods export tax, the more serious the trade war will be. According to equation (54), it is known that $s_{1}^{*}+t_{12}^{*}=s_{2}^{*}+t_{21}^{*}$ and is equal to a constant greater than zero. First, when the final goods export tax $s_{1}^{*}$ or $s_{2}^{*}$ is less than $-\alpha \left(2\beta^{2}+\beta \gamma -4\gamma^{2}\right)/\left(22\beta^{3}-25\beta^{2}\gamma -14\beta \gamma^{2}+16\gamma^{3}\right)$, Then $t_{12}^{*}>0$ or $t_{21}^{*}>0$. Then the trade war $t_{12}^{*}$ or $t_{21}^{*}$ will be bigger. Secondly, when the final goods export tax $s_{1}^{*}$ or $s_{2}^{*}$ is smaller, the trade war $t_{12}^{*}$ or $t_{21}^{*}$ is bigger. Q.E.D.

**Proposition 2** When the relationship between the self-price coefficient of demand and the cross-price coefficient of demand is $\beta >\left(-1+\sqrt{33}\right)\gamma /4$ or $\beta <\left(3+\sqrt{1945}\right)\gamma /44$, the trade war will not occur. Instead, both countries will subsidize each other's imports, fostering a more amicable trade relationship. A higher export tax on final goods will result in stronger reciprocity between country 1 and country 2. In essence, a higher $s_{1}^{*}$ ($s_{2}^{*}$) will lead to a lower $t_{12}^{*}$ ($t_{21}^{*}$).

Proof.

(1) This item will prove that when $\beta >\left(-1+\sqrt{33}\right)\gamma /4$ or $\beta <\left(1+\sqrt{145}\right)\gamma /12$, it will make $s_{3}^{*}=0$. According to the sixth item of Proposition 1, it has been proved: First, if $\beta <\left(1+\sqrt{145}\right)\gamma /12$, since $q_{1}=q_{2}=q_{3}>0$, the denominator of quantity is $22\beta^{3}-25\beta^{2}\gamma -14\beta \gamma^{2}+16\gamma^{3}<0$, and because $\beta <\left(1+\sqrt{145}\right)\gamma /12=1.0868\gamma$, so $\beta <\left(-1+\sqrt{33}\right)\gamma /4=1.18614\gamma$. Then $s_{3}^{*}<0$ is obtained from equation (54), so the contradiction holds. Secondly, when $\beta >\left(-1+\sqrt{33}\right)\gamma /4$, since $q_{1}=q_{2}=q_{3}>0$, the denominator of quantity is $22\beta^{3}-25\beta^{2}\gamma -14\beta \gamma^{2}+16\gamma^{3}>0$, and because $\beta >\left(-1+\sqrt{33}\right)\gamma /4$. Then $s_{3}^{*}<0$ is obtained from equation (54), so the contradiction holds. Since $s_{3}^{*}\geq 0$ is known, $s_{3}^{*}$ must be zero, that is, $s_{3}^{*}=0$. Since $\beta <\left(3+\sqrt{1945}\right)\gamma /44=1.0705\gamma <\left(1+\sqrt{145}\right)\gamma /12=1.0868\gamma$, so $s_{3}^{*}=0$ is also established. The proof steps for the second and third items below are the same as those for the first and second items in Proposition 1.
(2) This item will prove that equations (12), (13), (14) are as follows. Substitute equations (9), (10), (11) into equations (6), (7), (8), respectively, to obtain $\pi_{1}=\left[\beta p_{1}-\alpha -\gamma \left(p_{2}+p_{3}\right)\right]\left(a_{1}+a_{2}+a_{3}-p_{1}+s_{1}+t_{12}\right)$, and $\pi_{2}=\left(\beta p_{2}-\alpha -\gamma \left(p_{1}+p_{3}\right)\right)\left(a_{1}+a_{2}+a_{3}-p_{2}+s_{2}+t_{21}\right)$, and $\pi_{3}=\left(\beta p_{3}-\alpha -\gamma \left(p_{1}+p_{2}\right)\right)\left(a_{1}+a_{2}+a_{3}-p_{3}+s_{3}\right)$. From the first-order necessary conditions $\partial \pi_{1}/\partial p_{1}=0$, and $\partial \pi_{2}/\partial p_{2}=0$, and $\partial \pi_{3}/\partial p_{3}=0$. When the above three equations are combined, equations (12), (13), (14) are obtained.
(3) This item will prove that equation (15) is as follows. Bring the results of equations (12), (13), (14) back to equations (9), (10), (11), and then substitute equations (9), (10), (11) back to equation (5), the three equations of $\Omega_{1}$, $\Omega_{2}$, and $\Omega_{3}$ are obtained respectively. $\Omega_{1}=\beta a_{1}\left[3\alpha -\left(\beta -2\gamma \right)\left(3a_{1}+3a_{2}+3a_{3}+s_{1}+s_{2}+s_{3}+t_{12}+t_{21}\right)\right]/\left[2\left(\beta -\gamma \right)\right]$, and $\Omega_{2}=\beta a_{2}\left[3\alpha -\left(\beta -2\gamma \right)\left(3a_{1}+3a_{2}+3a_{3}+s_{1}+s_{2}+s_{3}+t_{12}+t_{21}\right)\right]/\left[2\left(\beta -\gamma \right)\right]$, and $\Omega_{3}=\beta a_{3}[3\alpha -\left(\beta -2\gamma \right)\left(3a_{1}+3a_{2}+3a_{3}+s_{1}+s_{2}+s_{3}+t_{12}+t_{21}\right)/\left[2\left(\beta -\gamma \right)\right]$. From the first-order necessary conditions $\partial \Omega_{1}/\partial a_{1}=0$, and $\partial \Omega_{2}/\partial a_{2}=0$, and $\partial \Omega_{3}/\partial a_{3}=0$. When the above three equations are combined, the first equation (15) is obtained, which is the general equation of the three equations.
(4) This item will prove that equations (16), (17), (18) are as follows. Bring back the result of the general equation of equation (15) to equations (2), (3), (4), (5), (6), (7), (8), (9), (10), (11), (12), (13), (14), and these results are substituted back into the first equation (1), then the three equations $W_{1}$, $W_{2}$ and $W_{3}$ are obtained respectively. The contents of $W_{1}$, $W_{2}$, and $W_{3}$ are too long, so they will not be listed in detail here. From the first-order necessary conditions $\partial W_{1}/\partial s_{1}=0$, and $\partial W_{2}/\partial s_{2}=0$, and $\partial W_{3}/\partial s_{3}=0$, and $\partial W_{1}/\partial t_{12}=0$, and $\partial W_{2}/\partial t_{21}=0$, and the proof of the first item has obtained $s_{3}^{*}=0$. When the above five equations are combined, equations (19), (20), (21) are obtained. They are as follows (i.e., equations (58), (59), (60)):

(58) $$s_{1}^{*}+t_{12}^{*}=-\frac{3\alpha \left(2\beta +\gamma \right)\left(2\beta^{2}+\beta \gamma -4\gamma^{2}\right)}{2\left(76\beta^{4}-56\beta^{3}\gamma -87\beta^{2}\gamma^{2}+44\beta \gamma^{3}+20\gamma^{4}\right)}$$

(59) $$s_{2}^{*}+t_{21}^{*}=-\frac{3\alpha \left(2\beta +\gamma \right)\left(2\beta^{2}+\beta \gamma -4\gamma^{2}\right)}{2\left(76\beta^{4}-56\beta^{3}\gamma -87\beta^{2}\gamma^{2}+44\beta \gamma^{3}+20\gamma^{4}\right)}$$

(60) $$s_{3}^{*}=0$$

(5) This item will prove the sign of the denominator $2\left(76\beta^{4}-56\beta^{3}\gamma -87\beta^{2}\gamma^{2}+44\beta \gamma^{3}+20\gamma^{4}\right)$ of equations (58), (59), (60). If $\beta >\left(3+\sqrt{1945}\right)\gamma /44$, then $2\left(76\beta^{4}-56\beta^{3}\gamma -87\beta^{2}\gamma^{2}+44\beta \gamma^{3}+20\gamma^{4}\right)>0$; if $\beta <\left(3+\sqrt{1945}\right)\gamma /44$, then $2\left(76\beta^{4}-56\beta^{3}\gamma -87\beta^{2}\gamma^{2}+44\beta \gamma^{3}+20\gamma^{4}\right)<0$. Bring the results obtained in equations (55), (56), (57), and the above four items back to equations (9), (10), (11), then the following equations (61), (62) are obtained.

$$q_{1}=q_{2}=\frac{\alpha \beta \left(2\beta +\gamma \right)\left(22\beta^{2}-3\beta \gamma -22\gamma^{2}\right)}{4\left(76\beta^{4}-56\beta^{3}\gamma -87\beta^{2}\gamma^{2}+44\beta \gamma^{3}+20\gamma^{4}\right)}$$

(61) $$=\frac{\;11\alpha \beta \left(2\beta +\gamma \right)\left[\beta +\left(-3+\sqrt{1945}\right)\gamma /44\right]\left[\beta -\left(3+\sqrt{1945}\right)\gamma /44\right]}{2\left(76\beta^{4}-56\beta^{3}\gamma -87\beta^{2}\gamma^{2}+44\beta \gamma^{3}+20\gamma^{4}\right)}$$

(62) $$q_{3}=\frac{\alpha \beta \left(32\beta^{3}-2\beta^{2}\gamma -29\beta \gamma^{2}+2\gamma^{3}\right)}{4\left(76\beta^{4}-56\beta^{3}\gamma -87\beta^{2}\gamma^{2}+44\beta \gamma^{3}+20\gamma^{4}\right)}$$

Because $q_{1}=q_{2}>0$, and because $\beta +\left(-3+\sqrt{1945}\right)\gamma /44>0$. If $\beta >\left(3+\sqrt{1945}\right)\gamma /44$, then $2\left(76\beta^{4}-56\beta^{3}\gamma -87\beta^{2}\gamma^{2}+44\beta \gamma^{3}+20\gamma^{4}\right)>0$ in the denominator of quantity can be obtained; If $<\left(3+\sqrt{1945}\right)\gamma /44$, then $2\left(76\beta^{4}-56\beta^{3}\gamma -87\beta^{2}\gamma^{2}+44\beta \gamma^{3}+20\gamma^{4}\right)<0$ in the denominator of quantity can be obtained.

(6) This item will prove that both equations (58), (59) are less than 0. Rewrite equations (58), (59) as equation (63).

$$s_{1}^{*}+t_{12}^{*}=s_{2}^{*}+t_{21}^{*}=-\frac{3\alpha \left(2\beta +\gamma \right)\left(2\beta^{2}+\beta \gamma -4\gamma^{2}\right)}{2\left(76\beta^{4}-56\beta^{3}\gamma -87\beta^{2}\gamma^{2}+44\beta \gamma^{3}+20\gamma^{4}\right)}$$

(63) $$=\frac{-3\alpha \left(2\beta +\gamma \right)\left[\beta +\left(1+\sqrt{33}\right)\gamma /4\right]\left[\beta -\left(-1+\sqrt{33}\right)\gamma /4\right]}{\left(76\beta^{4}-56\beta^{3}\gamma -87\beta^{2}\gamma^{2}+44\beta \gamma^{3}+20\gamma^{4}\right)}$$

It is known from the fifth item that if $\beta >\left(3+\sqrt{1945}\right)\gamma /44$, then $2\left(76\beta^{4}-56\beta^{3}\gamma -87\beta^{2}\gamma^{2}+44\beta \gamma^{3}+20\gamma^{4}\right)>0$; If $\beta <\left(3+\sqrt{1945}\right)\gamma /44$, then $2\left(76\beta^{4}-56\beta^{3}\gamma -87\beta^{2}\gamma^{2}+44\beta \gamma^{3}+20\gamma^{4}\right)<0$. First, if $\beta >\left(3+\sqrt{1945}\right)\gamma /44$, and $\beta >\left(-1+\sqrt{33}\right)\gamma /4=1.18614\gamma >\left(3+\sqrt{1945}\right)\gamma /44=1.0705\gamma$, so $s_{1}^{*}+t_{12}^{*}=s_{2}^{*}+t_{21}^{*}<0$. Second, when $\beta <\left(3+\sqrt{1945}\right)\gamma /44$, and $\beta <\left(3+\sqrt{1945}\right)\gamma /44=1.0705\gamma <\left(-1+\sqrt{33}\right)\gamma /4=1.18614\gamma$, so $s_{1}^{*}+t_{12}^{*}=s_{2}^{*}+t_{21}^{*}<0$. Q.E.D.

Proposition 3 When the relationship between the self-price coefficient of demand and the cross-price coefficient of demand is $\left(3+\sqrt{1945}\right)\gamma /44<\beta <\left(1+\sqrt{145}\right)\gamma /12$, the trade war is likely to occur. Any decrease in the export tax on final goods will further exacerbate the trade war. In essence, a reduction $s_{1}^{*}$ ($s_{2}^{*}$) will result in an increase in $t_{12}^{*}$ ($t_{21}^{*}$).

Proof.

(1) When $\beta <\left(1+\sqrt{145}\right)\gamma /12$ is known from the proof of the first item of Proposition 2, then $s_{3}^{*}=0$ will be obtained.
(2) From the proof of the fifth item of Proposition 2, when $\beta >\left(3+\sqrt{1945}\right)\gamma /44$ is known, then $2\left(76\beta^{4}-56\beta^{3}\gamma -87\beta^{2}\gamma^{2}+44\beta \gamma^{3}+20\gamma^{4}\right)>0$.
(3) From equation (57) in the proof of the sixth item of Proposition 2, and because $\beta <\left(1+\sqrt{145}\right)\gamma /12=1.0868\gamma <\left(-1+\sqrt{33}\right)\gamma /4=1.18614\gamma$, therefore, From equation (63), $s_{1}^{*}+t_{12}^{*}=s_{2}^{*}+t_{21}^{*}>0$ can be obtained. Q.E.D.

Proposition 4 Regardless of whether the trade war is caused by $\left(3+\sqrt{1945}\right)\gamma /44<\beta <\left(-1+\sqrt{33}\right)\gamma /4$ or trade subsidies resulting from $\beta >\left(-1+\sqrt{33}\right)\gamma /4$ or $\beta <\left(3+\sqrt{1945}\right)\gamma /44$, the equilibrium solutions for the production output of the three countries $q_{i}^{*}$, final goods price $p_{i}^{*}$, profit of final goods firm $\Pi_{i}^{*}$, intermediate goods price $a_{i}^{*}$, profit of intermediate goods firm $\Omega_{i}^{*}$, government tax $T_{i}^{*}$, and social welfare $W_{i}^{*}$ remain unaffected by the scale of the trade war or subsidy.

Proof.

(1) When $\left(1+\sqrt{145}\right)\gamma /12<$ $\beta <\left(-1+\sqrt{33}\right)\gamma /4$, a trade war may occur. Substituting equations (16), (17), (18) into equations (1), (2), (3), (4), (5), (6), (7), (8), (9), (10), (11), (12), (13), (14), (15), and equations (25), (26), (27), (28), (29), (30), (31) can be obtained, respectively.
(2) When $\left(3+\sqrt{1945}\right)\gamma /44<\beta <\left(1+\sqrt{145}\right)\gamma /12$, a trade war may occur. Bring equations (22), (23), (24) into equations (1), (2), (3), (4), (5), (6), (7), (8), (9), (10), (11), (12), (13), (14), (15), then the above equations (32), (33), (34), (35), (36), (37), (38), (39), (40), (41), (42), (43) can be obtained, respectively.
(3) When $\beta >\left(-1+\sqrt{33}\right)\gamma /4$ or $\beta <\left(3+\sqrt{1945}\right)\gamma /44$, no trade war will occur. Substituting equations (19), (20), (21) into equations (1), (2), (3), (4), (5), (6), (7), (8), (9), (10), (11), (12), (13), (14), (15) above, equations (32), (33), (34), (35), (36), (37), (38), (39), (40), (41), (42), (43) can be obtained, respectively.
(4) From the proofs of the above three items, whether it is equations (25), (26), (27), (28), (29), (30), (31) or equations (32), (33), (34), (35), (36), (37), (38), (39), (40), (41), (42), (43), they are all irrelated to the variables $t_{12}^{*}$ and $t_{21}^{*}$ of international trade tariffs or trade subsidies. Q.E.D.
